# Physiological and Behavioral Responses of Dairy Cattle to the Introduction of Robot Scrapers

**DOI:** 10.3389/fvets.2016.00106

**Published:** 2016-11-30

**Authors:** Renate L. Doerfler, Christina Lehermeier, Heike Kliem, Erich Möstl, Heinz Bernhardt

**Affiliations:** ^1^Agricultural Systems Engineering, TUM School of Life Sciences Weihenstephan, Technical University of Munich, Freising, Germany; ^2^Plant Breeding, TUM School of Life Sciences Weihenstephan, Technical University of Munich, Freising, Germany; ^3^Animal Physiology and Immunology, TUM School of Life Sciences Weihenstephan, Technical University of Munich, Freising, Germany; ^4^Unit of Physiology, Pathophysiology and Experimental Endocrinology, University of Veterinary Medicine of Vienna, Vienna, Austria

**Keywords:** heart rate variability, behavior, cortisol metabolites, stress, habituation, sensitization, robot scraper, dairy cow

## Abstract

Autonomous mobile robot scrapers are increasingly used in order to clean the floors on dairy farms. Given the complexity of robot scraper operation, stress may occur in cows due to unpredictability of the situation. Experiencing stress can impair animal welfare and, in the long term, the health and milk production of the cows. Therefore, this study addressed potential stress responses of dairy cattle to the robot scraper after introducing the autonomous mobile machine. Thirty-six cows in total were studied on three different farms to explore possible modifications in cardiac function, behavior, and adrenocortical activity. The research protocol on each farm consisted of four experimental periods including one baseline measurement without robot scraper operation followed by three test measurements, in which cows interacted with the robotic cleaning system. Interbeat intervals were recorded in order to calculate the heart rate variability (HRV) parameter RMSSD; behavior was observed to determine time budgets; and fecal samples were collected for analysis of the cortisol metabolites concentration. A statistical analysis was carried out using linear mixed-effects models. HRV decline immediately after the introduction of the robot scraper and modified behavior in the subsequent experimental periods indicated a stress response. The cortisol metabolites concentration remained constant. It is hypothesized that after the initial phase of decrease, HRV stabilized through the behavioral adjustments of the cows in the second part of the study. Persistent alterations in behavior gave rise to the assumption that the animals’ habituation process to the robot scraper was not yet completed. In summary, the present study illustrated that the cows showed minor signs of disturbance toward the robotic cleaning system. Thus, our findings suggest that dairy cattle can largely adjust their behavior to avoid aversive effects on animal welfare. Additional research can provide further insight into the development of the animal–machine interaction beyond the initial phase of robot scraper operation considered in this study.

## Introduction

On dairy farms, the cleaning of walkways is crucial to ensure proper hygienic conditions and claw health in cows. Recently introduced mobile robot scrapers complete about 15 cleaning procedures on different routes daily, each taking approximately 35 min. The complexity of robot scraper operation can potentially reduce the predictability of the cows’ situation. Unpredictability of circumstances is known to arouse stress responses in animals (1, 2). Experiencing prolonged stress can be associated with a decline in immune competence, health status, and milk production in dairy cattle (3, 4).

Stress is a bodily response to stimuli (stressors), which can originate from dominant conspecifics, anthropogenic stressors, or the technical environment. Regulatory mechanisms of the body maintain stability by behavioral and physiological adjustments (5). On the one hand, the adverse external stimulus can activate the sympathetic–adrenal medulla (SA) axis of the autonomic nervous system and induce an increase in heart rate (HR), secretion of catecholamines including adrenaline and noradrenaline, vascular pressure, and a reduction of gastrointestinal activity. On the other hand, it can activate the hypothalamic–pituitary–adrenal cortex (HPA) axis that stimulates the release of adrenocorticotropic hormone (ACTH) from the anterior pituitary gland and of glucocorticoids, such as cortisol, from the adrenal cortex (3).

Novelty of a stimulus enhances its impact on animals. Stress responses to a novel object range from alertness associated with curiosity to a state of alarm and persistent interest (5). Since the stress response is closely related to the animals’ perception and their capability to act against the stressor, novel stimuli affect not only the physiological but also the emotional state (4). They may not be experiencing stress, if the potential stressor permits animals to anticipate the future circumstances and to adjust their physiology and behavior to the stimulus (1).

Exposure to a repeated stressful stimulus often results in attenuated responsiveness to the stressor imposed on the animal that is referred to as habituation. In this process of non-associative learning (6–8), large amounts of information from sensory input are filtered and the synaptic transmission of signals from the receptors to the brain and to effectors is reduced, when adverse consequences fail to emerge (6, 9, 10). Repeated exposure to a stressor can also induce a sensitization process in animals. Sensitization – the enhancement of a response to a repeated stimulus (11) – is attributed to a vast range of stimuli, whereas habituation is largely stimulus specific (12).

Stress responses can be understood as adaptive bodily reactions to ensure survival (5, 9). Short-term strategies (fight-or-flight responses) to perceived stressors adopted by animals comprise physiological adjustments and altering of behavior. In the long term, stimuli activate ACTH release from the pituitary, which increases production of glucocorticoids from the adrenocortical tissue (1).

Heart rate and heart rate variability (HRV) have previously been used to assess stress responses in farm animals (13–19) and indicate disturbances of the sympathovagal balance. Progressing gestation and physical activity result in an increase in HR and a decrease in HRV (18, 20–22). A number of previous studies have suggested that the stress response of the cardiac system mainly relies on parasympathetic activity (23–27), and therefore, HRV is considered a more suitable indicator to assess stress in animals than HR. A recent review identified growing evidence that higher vagal tone in individuals under resting conditions is associated with higher resilience to stress (22).

Alterations in behavior frequently indicate a stress response and can result in behavioral disorders when stress is severe. Animals attempt to counteract aversive environmental stimuli by behavioral alterations (4). A number of studies have been carried out to investigate changes in animal behavior as an indicator of stress (28–30). In sows over the course of gestation, inactivity increased, whereas walking, standing, and rooting decreased (18, 19).

In order to assess stress, glucocorticoid levels have often been considered in conjunction with the parameters of cardiac activity and animal behavior. Recent studies confirmed the validity of elevated levels of adrenocortical activity for the analysis of stress responses in dairy cattle (30–32). Measuring cortisol metabolites in fecal samples avoids invasive sampling techniques (venipuncture) that may have an influence on circulating hormone levels. Cortisol is almost completely metabolized when excreted *via* feces. Fecal samples characterize the stress level in dairy cows 10–12 h prior to sampling, which represents the transfer time through the intestinal tract (33, 34).

A novel moving object such as a robot scraper may induce stress in cows. Unlike manure scrapers moving back and forth along the entire walkway width, autonomous mobile robot scrapers clean the floor at a speed varying between 4 and 18 m/min. A slide is either mounted on the front of the housing with a working width of 1.00–2.10 m or below the housing. Robot scrapers are guided along the walkways and crossovers either by sensors or by passive transponders embedded in the floor surface. During an initial learning session, different routes across the barn design are programed and then followed by the robot automatically. Robot scraper operation is interrupted for about six consecutive hours a day for the charging of the accumulators. If the robot scraper encounters a cow, it attempts to bypass the animal several times before switching itself off.

Only a few previous works investigated the impact of manure scrapers on stress reactions in dairy cows. Buck et al. (14) in their study identified a slight stress response in cows during scraping events indicated by a decrease of the HRV. The animals also shifted feeding from the daytime toward the nighttime and increased the number of their feeding bouts when the manure scraper moved along the feeding fence shortly after feed provision. Neither the scraper type nor scraping besides the usual times affected the avoidance reactions of the cows. Another previous study observed stumbling both in moving cows who encountered the manure scraper and in cows standing in a group who unexpectedly encountered the moving scraper. In the majority of cases (81 of 107), the hind legs of the cow were involved (35).

How the use of autonomous mobile cleaning systems affects dairy cows has not yet been examined. The objective of this study therefore was to explore potential stress responses in cows during the interaction with robot scrapers. Cardiac activity, animal behavior, and the cortisol metabolites concentration in feces were used as indicators of the response.

## Materials and Methods

This study was carried out in accordance with the recommendations of the German Animal Welfare Act. The research protocol was approved and supervised by the animal care officer of the TUM School of Life Sciences Weihenstephan, Technical University of Munich, and the veterinary office of the district of Freising, Bavaria, Germany.

### Animals, Housing, and Robot Scrapers

This study was conducted from January to July 2013 to identify possible effects of robot scraper operation on animal physiology and behavior. A total of 36 lactating cows were observed on 3 different farms: the experimental farm of the TUM School of Life Sciences Weihenstephan, Technical University of Munich, Bavaria, Germany (farm 1); the Teaching, Research and Demonstration Center Kringell, Bavaria, Germany (farm 2); and a commercial dairy farm in Bavaria, Germany (farm 3). From each farm, 12 focal animals were randomly selected out of the herd of the lactating cows. Their mean age was 5 years (min 2; max 8) with more than 60% of the animals aged between 2 and 4 years. The stage of lactation was divided into early lactation (lactation months 1–4), mid lactation (lactation months 5–8), and late lactation (lactation months >8). The percentage of cows was 38.9 (*n* = 14) in early lactation, 44.4 (*n* = 16) in mid-lactation, and 16.7 (*n* = 6) in late lactation. Daily milk yield of the cows at the beginning of the experiment was 22.2% in the category of ≤19 kg, 30.6% in the category of 20–29 kg, 36.1% in the category of 30–39 kg, and 11.1% in the category of >40 kg.

The three dairy herds involved in this study were kept in loose housing systems on slatted floors. On farm 1, the 46 lactating Brown Swiss cows were housed in a barn comprising 2 compartments (437 m^2^) situated on both sides of the central feeding alley. One compartment consisting of two subareas had dimensions 13.80 m × 10.30 m and 4.40 m × 7.30 m. The other compartment also consisting of two subareas had dimensions 24.70 m × 10.30 m and 4.00 m × 2.20 m. Both compartments were equipped with two parallel rows of deep bed cubicles and feeding places, where at least one cubicle and one feeding place were available per cow. The walkways between cubicles were 2.50 m in width and those at the feeding fence were 3.00 m in width. Rubber mats covered the slatted floors. During the study, the 12 focal animals selected out of the lactating cows moved around both compartments, which were connected *via* a foldable bridge interrupting the feeding fence.

On farm 2, the 62 lactating Simmental cows were kept in one compartment at one side of the central feeding alley consisting of two subareas, which had dimensions 35.00 m × 11.85 m and 10.00 m × 2.88 m, a total of 443 m^2^. The compartment included three rows of deep bed cubicles parallel to the feeding alley. There was a small walkway between cubicles with a width of 1.90 m and a broader walkway at the feeding fence with a width of 2.80 m. All walking areas were equipped with rubber mats. The cows had access to concentrate dispensers in order to supplement their concentrate ration. Drinkers and brushes were positioned in the crossover zones connecting the main walkways. The 12 selected focal cows were observed in the compartment where the lactating animals were housed.

The barn design on farm 3 also comprised two compartments on both sides of the central feeding alley. All the 12 focal cows were selected from the high-yielding group of the Simmental herd consisting of 91 animals and were housed in 1 of the compartments. The dimensions of this compartment were 60.00 m × 14.70 m, encompassing an area of 882 m^2^. It was furnished with high bed cubicles with soft rubber mats and a thin layer of sawdust renewed weekly. The walkway between cubicles was 3.00 m in width and the walkway at the feeding fence 4.00 m in width. Concentrate feed was allocated in concentrate dispensers. Drinkers and brushes were placed in the crossovers.

**Figure 1:**
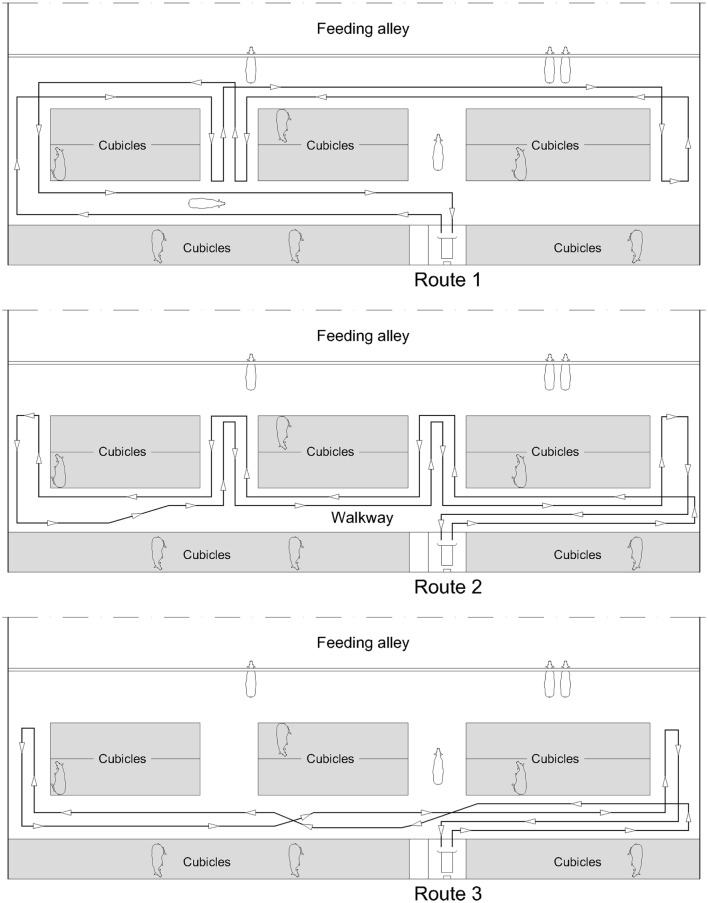
**Scheme of the three routes of the scraper robot in farm 3, rotating throughout each day**.

The robot scraper (DeLaval, Glinde, Germany; RS420) used on all three farms was 1.48 m in length, 0.72 m in width, and 0.64 m in height. The slide in front of the body was 1.00 m (farms 1 and 2) or 1.30 m (farm 3), respectively. A quiet sound of the two electric motors was audible when the robot scraper was in operation. As illustrated in Figure 1, the robot scraper took different routes along the walkways. In the feeding area, the robot did not move directly along the feeding fence but behind the standing cows. On farm 1, however, the robot moved 5 m directly along the feeding fence. The working speed of the robots was 4.0 m/min (farms 1 and 2) or 5.5 m/min (farm 3), respectively. Cleaning the surfaces of main and connecting walkways, the robot scrapers had a daily running time of about 15 h. During the recharging of the accumulators, the robots did not run for approximately six consecutive hours a day.

### Experimental Protocol

The experimental protocol consisted of four experimental periods on each farm. One 1-week baseline measurement (B) prior to the implementation of the robot scraper was followed by three 1-week test measurements (T1, T2, T3) extended over a period of 4 weeks (Table 1). In week 3, there was no data collection. On each farm, a habituation period of 5 days was scheduled prior to the baseline measurement during which animals were accustomed to the handling procedure associated with data collection. During each experimental period, HRV was measured and cow behavior was observed for 5 days. Fecal samples were collected to analyze the cortisol metabolites concentration on days 2, 3, and 5 of each experimental period. Since fecal samples reflect the cortisol metabolites concentration in cows 10–12 h prior to sampling, the measurements refer to days 1, 2, and 4 of each experimental period. At least 1 day before initial operation, the robot scraper was placed in its parking area in the barn in order to allow animals to get accustomed to it.

**Table 1:** **Experimental protocol for data collection on three farms (*n* = 36) from January to July 2013**.

Experimental period	Robot operation	Animal handling	Farm		
			1	2	3
			
			(*n* = 12)	(*n* = 12)	(*n* = 12)
Habituation (H)	No	Yes	–	–	–
Baseline measurement (B)	No	Yes	HRV	HRV	HRV
			AB	AB	AB
			FMC	FMC	FMC
Test measurements (T1, 2, 3)	Yes	Yes	HRV	HRV	HRV
			AB	AB	AB
			FMC	FMC	FMC

*HRV, heart rate variability; AB, animal behavior; FMC, fecal cortisol metabolites*.

### Recording and Analysis of Cardiac Activity

A portable radio telemetric HR monitoring system (Polar Electro Oy, Kempele, Finland; Polar Equine RS800CX Science) was used to record the interbeat intervals of cows. This non-invasive system included an elastic chest belt with two integrated electrodes that measured the electric voltage on the skin produced by the heart, a wrist-watch receiver for data storage that was attached to the outside of the belt in a small plastic case, and a transmitter for wireless transmission of HR signals to the receiver. The validity of this system was recently verified by Essner et al. (36).

The HR monitor was fitted around the thorax of the animal right behind the scapula, and the electrodes were placed on the left side of the cows: one dorsal behind the scapula and the other ventral behind the forelimb. To ensure proper transmission of signals between skin and electrodes, cows were shaved where the electrodes were positioned and a layer of electrode gel was applied to the electrode surfaces every day. An additional belt was fitted over the HR monitor to secure it in the required position. After evening milking, the correct fit of the chest belt was checked and, if necessary, adjusted. Since the storage capacity of receivers was restricted, after the morning milking, data were downloaded onto a notebook using a serial interface. The wrist-watch receivers of the HR monitors were synchronized with the video cameras. A team consisting of six persons handled the animals from habituation to the end of the experiment.

As recommended by the Task Force of the European Society of Cardiology and the North American Society of Pacing and Electrophysiology (21), the different steps undertaken in the analysis of HRV were the selection of interbeat intervals over periods of 5 min, error correction of the numerically stored interbeat intervals, and the calculation of the HRV parameter RMSSD. The analysis of the HRV was carried out on days 2 and 4 of each experimental period (B, T1, T2, T3). During these experimental periods, interbeat intervals over 5-min periods were considered in all 36 cows both during activity (feeding, walking) and at rest, to minimize the influence of varying physical activity on cardiac function. For physically active cows, the interbeat intervals were selected within the period from noon to 3:00 p.m. and for resting cows from 9:00 p.m. to midnight.

In order to determine effects of the proximity of the moving robot scraper on the HRV of lying cows within T1, T2, and T3, interbeat intervals over 5-min periods were selected from 10:00 a.m. to 3:00 p.m. on two variable days of each experimental period. The interbeat intervals were analyzed 2.5 min prior to and 2.5 min after the distance between the moving robot scraper and the animal reached a minimum (near) or a maximum (far), respectively. Interbeat intervals were merely included in the analysis when the respective behavior of the cows had continued for at least 5 min.

Failure of measurement and possibly errors could have occurred when the belts of the monitoring system moved out of place and the contact area between electrodes and animal was poor. Error correction of interbeat intervals was performed with the correction procedure of the software of the monitoring system (Polar Electro Oy, Kempele, Finland; Polar ProTrainer 5 Equine Edition, Version 5.35.161). Only those 5-min periods were used that met a corrected error rate of less than 5% and did not contain three or more subsequent erroneous interbeat intervals, as suggested by von Borell et al. (23). Cardiac data were visually inspected, and anomalies were identified based on the five error categories described by Marchant-Forde et al. (18). In addition, the extent of successive interbeat intervals was examined mathematically using the error identification procedure according to Cheung (37). The interbeat interval in question was considered erroneous, and error correction was performed, if any interval was 20% greater or smaller than the preceding interval.

Type 1 errors including one deviating interval were corrected by replacing the erroneous interval using linear interpolation. Types 2 and 3 errors consisted of two immediately consecutive intervals containing errors: the first being 20% wider than the preceding interval and the second being 20% narrower than the following interval or *vice versa*. Correction of types 2 and 3 errors was carried out by merging the two successive erroneous intervals and by dividing the sum of both intervals by 2. Type 4 errors including markedly too wide intervals were corrected by subdividing the erroneous interval into two intervals. The correction of type 5 errors (two successive too narrow intervals) was carried out by merging the two erroneous intervals into one interval.

Characterizing the variation in the interval between consecutive heart beats (21), the evaluation of HRV was based on the time-domain analysis of interbeat intervals and the associated quantification of the root mean square of successive interbeat interval differences (RMSSD, milliseconds). In order to take account of the influence of physical activity in cows, RMSSD were calculated during activity (RMSSD_a_), and at rest (RMSSD_r_).

### Observation and Analysis of Behavior

Animal behavior was recorded using 14 (farms 1 and 2) or 16 (farm 3) video cameras with integrated infrared illuminators (Mobotix, Langmeil, Germany; Mobotix MX-D14Di-Sec D22N22) for records during day and nighttime. Continuous recording was carried out based on the experimental design. The behavioral protocol addressed the times cows spent lying, feeding, standing in total, standing in the cubicle, standing in the walking area, and locomotion of the animals (Table 2). Feeding independently of the actual feed intake was identified by observing whether the animals had their heads above the feeding trough.

**Table 2:** **List of time budget parameters and their definition**.

Behavior	Definition
Lying	Lying in the sternal or side position including lying down
Standing in total	Standing in an upright position including standing up
Standing in the cubicle	Standing in an upright position in the cubicle (at least with one foot) including standing up
Standing in the walking area	Standing in an upright position in the walking area including standing up
Feeding	Feeding at the feeding place with the cow’s head above the feeding trough
Locomotion	Locomotion moving forward or backward in the walking area at pace, trot, and gallop

Video data were analyzed by means of the evaluation software (Mangold International, Arnstorf, Germany; INTERACT^®^). On days 2 and 4 of each experimental period (B, T1, T2, T3), daily time budgets of the different behavioral parameters were analyzed from midnight to 4:00 a.m., 10:00 a.m. to 3:00 p.m., and 7:00 p.m. to midnight. These time budgets excluded the times of milking and of measurement procedures.

### Fecal Sampling, Extraction, and Analysis

Fecal samples for the analysis of cortisol metabolites 11,17-dioxoandrostanes (11,17-DOA) concentrations were collected rectally from each animal between 7:30 a.m. and 10:30 a.m. The samples were stored in 50-ml falcon tubes (Sarstedt, Nürnbrecht, Germany), immediately cooled by freezer packs, and within 3 h after collection frozen at −20°C until assay.

The frozen fecal samples were thawed for 30 min at room temperature. The 0.5-g wet feces of each sample was vortexed on a multi-vortex (VWR International GmbH, Darmstadt, Germany; Tube Rotator) for 30 min with 5-ml 80% methanol (Avantor, Griesheim, Germany; J.T. Baker^®^ Chemicals) and centrifuged at 2,500 × *g* for 15 min at room temperature. The supernatant was pipetted into a tube and stored at −20°C until analysis. 11,17-DOA was analyzed using the group-specific 11-oxoetiocholanolone enzyme immunoassay (EIA) from Möstl and Palme (Labcode 72a, Department of Biomedical Sciences, Physiology, University of Veterinary Medicine, Vienna, Austria). Extraction and EIA were carried out as described by Palme and Möstl (38). Statistical analysis of 11,17-DOA concentration was carried out for days 2, 3, and 5 of each experimental period (B, T1, T2, T3).

### Statistical Analysis

In the assessment of cardiac data during baseline and test measurements, the analysis of RMSSD_a_ was based on a total of 216 valid measurements (*n* = 58, *n* = 58, *n* = 49, *n* = 51 in B, T1, T2, and T3, respectively). A total of 228 valid measurements formed the database for the analysis of RMSSD_r_ (*n* = 54, *n* = 56, *n* = 61, *n* = 57 in B, T1, T2, and T3, respectively). Either failure of the HR monitoring system or inappropriate behavior of cows in the scheduled times (feeding and walking from noon to 3:00 p.m.; lying from 9:00 p.m. to midnight) had led to missing data. The analysis of HRV relating to the distance between the robot and the cow involved 147 valid measurements in close proximity to the cow (*n* = 45, *n* = 50, *n* = 52 in T1, T2, and T3, respectively) and 149 valid measurements at a distance from the cow (*n* = 48, *n* = 51, *n* = 50 in T1, T2, and T3, respectively). A total of 273 valid behavioral observations were used for the analysis of the time budgets during the experimental periods B, T1, T2, T3. Behavioral information was missing due to a lack of video data.

The effect of the experimental period on cardiac activity, behavior, and 11,17-DOA concentration was analyzed by linear mixed-effects models using the lme4 package in R 3.2.2 (39). All mixed models included the factor variable experimental period as fixed effect and the farm as well as the cow (nested within farm) as random effects. In addition, the interaction term between cow and experimental period was considered as a random effect in the mixed model in order to account for individual changes in the cows over time, such as the progressing gestation. Differences between experimental periods were tested by t-tests. All differences with *P* values <0.05 were considered as significant. *P* values and SEs of the fixed effects were calculated using Satterthwaite approximations for the degrees of freedom within the lmerTest R package (40).

Moreover, the effect of the distance of the moving robot on the HRV of the lying cow was analyzed by linear mixed-effects models. The mixed model for HRV included the indicator variable for robot position (near, far), the experimental period, and the interaction between robot position and experimental period (T1, T2, and T3) as fixed effects and the farm, the cow (nested within farm), and the interaction between cow and experimental period as random effects.

Prior to analysis, the residuals of the models were graphically assessed for normal distribution and homogeneity of variances by quantile–quantile (q–q) plots. When the model assumptions were not met, data were transformed. To determine the impact of the robot scraper on the cows within all experimental periods, the analysis of HRV relied on double logarithmic transformation, 11,17-DOA concentration on logarithmic transformation and behavioral patterns related to standing were square root transformed. The analysis of HRV associated with robot proximity was based on double logarithmic transformation. Outlying observations with standardized residuals outside the 99% confidence interval of the standard normal distribution were removed. The maximal number of values removed was 8 for the parameter RMSSD_r_.

## Results

### Heart Rate Variability

**Figure 2:**
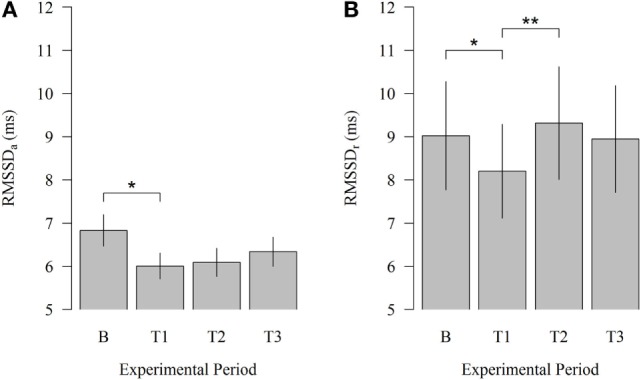
**Effects of the robot scraper on the heart rate variability of dairy cows**. **(A)** Root mean square of successive beat-to-beat differences (RMSSD) during physical activity and **(B)** at rest in the baseline measurement (B) and test measurements (T1, T2, T3). Vertical lines indicate SEMs and asterisks significant differences between means of the different experimental periods (*P < 0.05, **P < 0.01, ***P < 0.001). RMSSD_a_, RMSSD of cows during physical activity. RMSSD_r_, RMSSD of cows at rest.

Robot scraper operation significantly affected HRV of cows immediately after the introduction of the robot. As illustrated in Figure 2, HRV during physical activity (HRV_a_) decreased from B to T1 (6.83 vs. 6.01; *P* = 0.030). Moreover, HRV at rest (HRV_r_) significantly declined from B to T1 (9.02 vs. 8.20; *P* = 0.047) and increased from T1 to T2 (8.20 vs. 9.32; *P* = 0.007) when the robot scraper was used for walkway cleaning. HRV_a_ was higher than HRV_r_ during each experimental period corresponding to our expectations.

**Figure 3:**
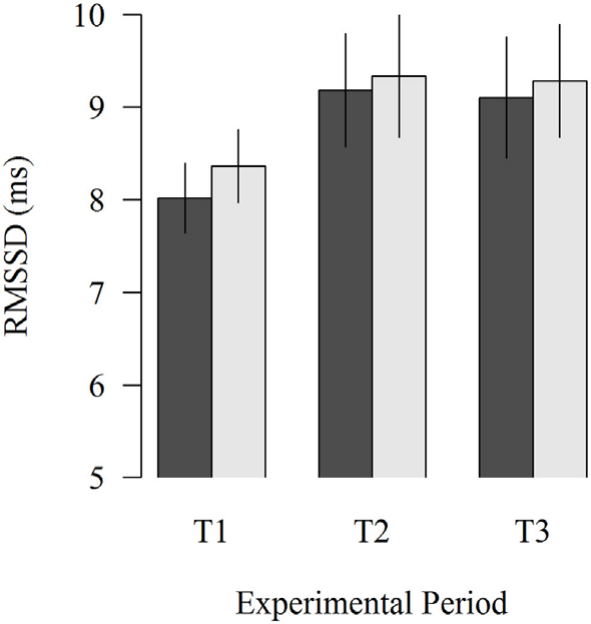
**Effects of the far (black column) and near (gray column) distance of the moving robot scraper on the HRV of lying cows during the experimental periods T1, T2, and T3**. Vertical lines indicate SEMs.

The study of the proximity of the moving robot scraper on the HRV of lying cows showed no significant effects (Figure 3). In all experimental periods, HRV was numerically lower when the moving robot scraper was far away from the cow than when it was close to the cow (8.02 vs. 8.36; *P* = 0.276, 9.18 vs. 9.34; *P* = 0.675, 9.10 vs. 9.29; *P* = 0.596 in T1, T2, and T3, respectively).

### Behavior

**Figure 4:**
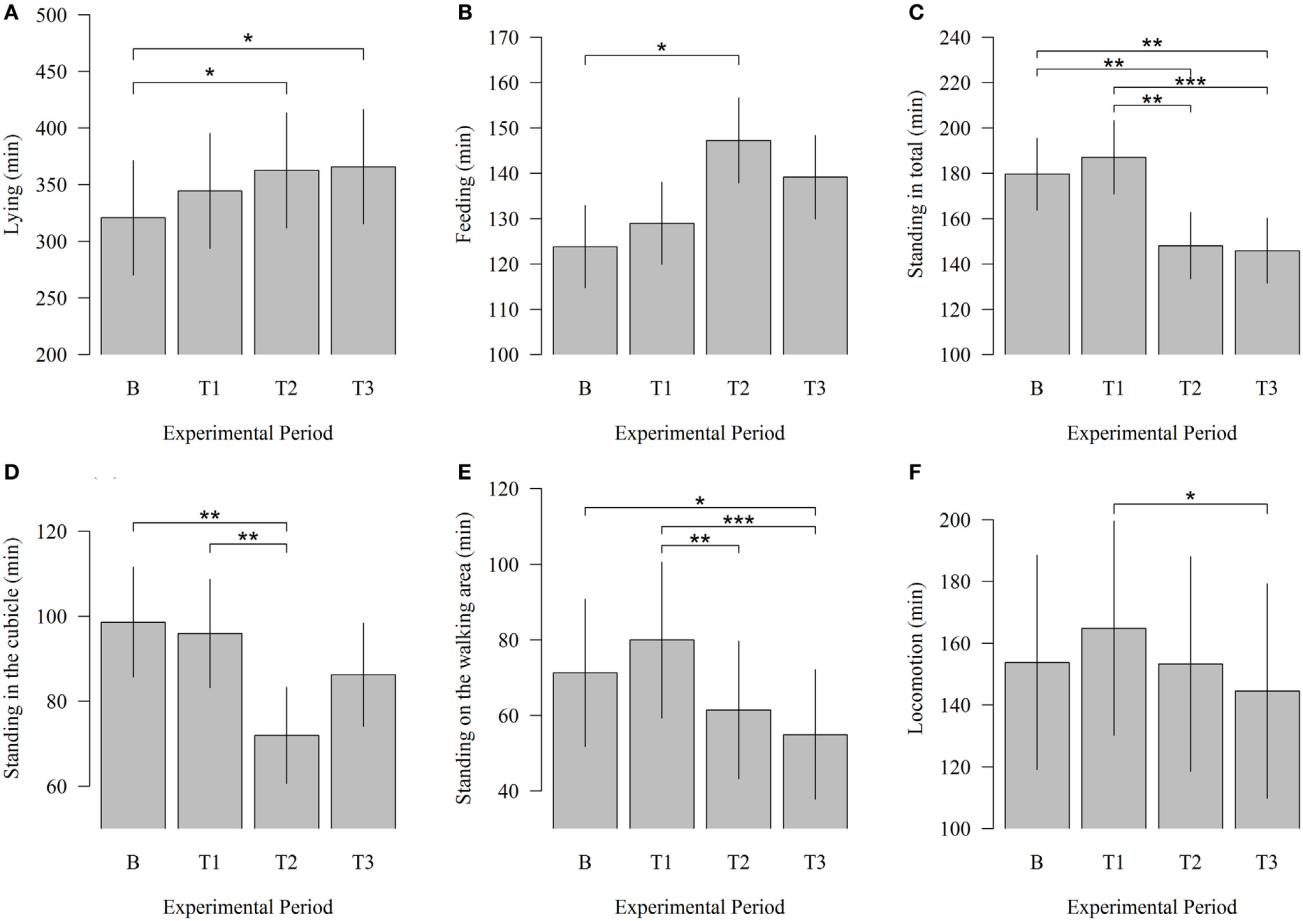
**Effects of the robot scraper on the behavior of dairy cows**. **(A)** Times cows spent lying, **(B)** feeding, **(C)** standing in total, **(D)** standing in the cubicle, **(E)** standing in the walking area, and **(F)** walking for the baseline measurement (B) and test measurements (T1, T2, T3) based on a daily observation time of 14 h. Vertical lines indicate SEMs and asterisks significant differences between means of the different experimental periods (*P < 0.05, **P < 0.01, ***P < 0.001).

In this study, potential changes in time budgets related to lying, feeding, standing, and walking between baseline and test measurements were examined. Modifications in behavioral patterns occurred beginning with T2. The time cows spent lying down significantly increased (Figure 4) between B and T2 (320.77 vs. 362.59; *P* = 0.023) and between B and T3 (320.77 vs. 365.79; *P* = 0.013). In T1 cows, lying down time was numerically higher than in B. The time cows spent lying reached a maximum in T3, 4 weeks after the introduction of the cleaning system. Robot scraper operation had also a significant influence on the time animals spent feeding. The animals spent significantly more time feeding during T2 than during B (147.28 vs. 123.83; *P* = 0.023).

Correspondingly, the time cows spent standing in total significantly decreased between B and T2 (179.63 vs. 148.06; *P* = 0.007) and between B and T3 (179.63 vs. 145.86; *P* = 0.004). Standing in total was numerically higher during B than during T1. When solely considering the test measurements, a significant decline can be identified between T1 and T2 (187.10 vs. 148.06; *P* = 0.001) and between T1 and T3 (187.10 vs. 145.86; *P* < 0.001). In this context, standing in the cubicle was reduced significantly between B and T2 (98.59 vs. 71.97; *P* = 0.003) and between T1 and T2 (95.92 vs. 71.97; *P* = 0.007). The time cows spent standing in the walking area significantly decreased between B and T3 (71.26 vs. 54.91; *P* = 0.013). Moreover, a comparison of the test measurements showed that the time animals spent standing in the walking area was significantly lower during T2 (61.43 vs. 71.26; *P* = 0.009) and T3 (54.91 vs. 71.26; *P* < 0.001) than during T1.

The locomotion among animals decreased significantly between T1 and T3 (164.83 vs. 144.59; *P* = 0.031) but showed no significant effects between baseline and test measurements.

### Concentration of Cortisol Metabolites

**Figure 5:**
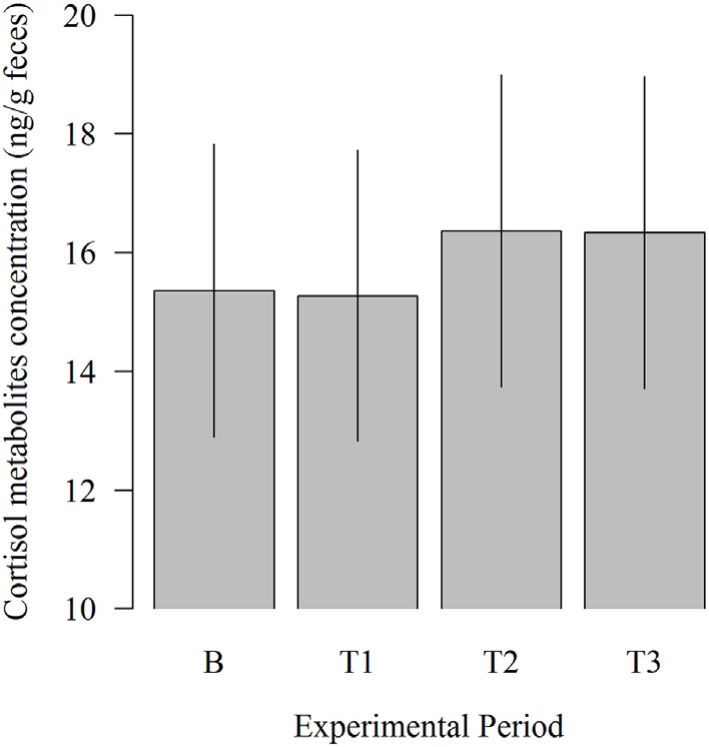
**Effects of the robot scraper on the fecal cortisol metabolites concentration of dairy cows for the baseline measurement (B) and test measurements (T1, T2, T3)**. Vertical lines indicate SEMs and asterisks significant differences between means of the different experimental periods (*P ≤ 0.05, **P ≤ 0.01, ***P ≤ 0.001).

There was no significant influence of the experimental period on fecal cortisol metabolites concentration (Figure 5). However, 11,17-DOA concentration was numerically higher in T2 (16.36 vs. 15.36; *P* = 0.309) and T3 (16.33 vs. 15.36; *P* = 0.333) compared to B.

## Discussion

### Heart Rate Variability

In this study, we explored the cows’ potential stress responses to the introduction of robot scrapers with respect to HRV. The significant decline in HRV between baseline measurement and test measurement 1 indicated that in both active and recumbent cows, the sympathetic nervous system was activated in response to the presence of the robot scraper. It can be reasonably assumed that during the first phase of robot scraper operation, novelty and alternating routes of the robot scraper induced a stress response in the cows. The fact that no significant differences were identified between baseline measurement and test measurements 2 and 3 provided evidence that, after an initial stress response, cows were able to effectively cope with the presence of the robot scraper.

Wingfield and Ramenofsky (1) suggested that the modification of behavior is vital for animals to successfully adjust to detrimental stimuli and to avoid stress. It is therefore very likely that the alterations in animal behavior during T2 and T3 had resulted in a successful coping process. Cows adjusted to the situation by changing location and by avoiding encounters with or close proximity to the robot. Individual differences in cows over the experimental periods were taken into consideration in the statistical model, thus accounting for the effects of the stage of gestation (18) or lactation on the stress responses.

The analysis of HRV in recumbent cows when the robot was at close proximity to or at a distance from the cow did not confirm the stress response identified between baseline measurement and test measurement 1. One reason for this could be that the two 5-min interbeat intervals (far and near) were usually selected within one robot scraper route during which several encounters between cow and machine occurred. Thus, the time distance between the two 5-min interbeat intervals was small, and possibly, the boundaries between “far” and “near” had become blurred. The lowest HRV in T1 and its tendency to increase in T2 and T3 corresponded with the results derived from the analysis of HRV during baseline and test measurements.

In agreement with our research, previous studies demonstrated that the exposure to aversive stimuli decreases HRV (41–43). The findings of Buck et al. (14) verified a minor stress response in cows during the scraping events of a manure scraper based on a decrease of the HRV, whereas studies examining the cow response to automatic milking systems found no indicators of stress (13, 29). In the present study, the significant declines of RMSSD_a_ and RMSSD_r_ from B to T1 and the significant elevation of RMSSD_r_ from T1 to T2 reaching the level of B seems to indicate that the cardiac function normalized in the presence of the robot scraper and habituation was successful. Diurnal variation in operating and charging periods of the robot may initiate sensitization and desensitization processes. However, the trends of RMSSD_a_ and RMSSD_r_ during the limited experimental period show only very weak signs of sensitization in cows.

Recent research explored short- and long-term effects of chronic stress on cardiac autonomic regulation. Rats showed continuous parasympathetic activation, an increase in the HRV parameter RMSSD or “enduring vagal rebound” after they were submitted to intermittent episodes of subordination associated with agonistic interaction (44, 45). According to Carnevali and Sgoifo (22), this temporary compensatory response initially enabled animals to successfully cope with the perceived sympathetic hyperactivity triggered by stressful conditions but may fail after long-term exposure to a stressor. Alternating periods of operation and non-operation (charging) in the robot scraper imply potential occurrence of an “enduring vagal rebound” in the present study. However, additional research over the experimental periods considered is necessary to confirm this hypothesis.

### Behavioral Changes

Besides the physiological parameters, time budgets related to lying, feeding, standing, and walking were examined in this study in order to identify potential stress responses in dairy cows. The analysis of baseline and test measurements showed that alterations in behavior did not take place instantaneously but with a time delay of approximately 1 week after the introduction of the robotic cleaning system. Possible effects of progressing gestation (18) on animal behavior were taken into account in the statistical model.

We assume that modifications in behavior occurred in order to re-establish stability of physiological function and to mitigate the effects of the persistent stressor on the cardiovascular system. Both physiological stability and cow health are at risk when adaptation to the challenge is unsuccessful (46). Behavioral adjustments are adopted by animals as a short-term strategy to overcome stressful conditions (1, 4, 44). Thus, the modified behavior showed by the cows provides evidence for some stress response. The temporary delay in the behavioral alteration appears to be a reasonable strategy for avoiding growing discomfort and energy exertion when the aversive stimulus is imposed on the animal for a prolonged period.

Furthermore, the animal’s capability to carry out effective behavioral action determines the harmfulness of a stressor (4). Leaving an area where the robot operates can terminate the negative impact of this stimulus and can help to adjust to the situation. In this context, alterations in behavior can prevent psychological stress in cows indicated by a decline in HRV during T1. The comparison between the time budgets in the baseline and test measurement periods demonstrated that animals reduced the time spent standing in the walking area, where the robot was in operation. On the other hand, cows spent more time lying and feeding, thus avoiding confrontations with the robot scraper. Buck et al. (14) confirmed alterations in animal behavior, such as an increase in feeding bouts and in nighttime feeding when the manure scraper moved along the feeding area shortly after feed supply.

The data of our research did not confirm our expectations that locomotion in conjunction with alarm and flight responses increases when the robot scraper was introduced. Although a significant change between B and T1 failed to appear, the significant decrease in the time cows spent walking between T1 and T3 is an indicator for minor modification in behavior. An explanation for the weak response in locomotor activity can be that the robot scraper allows cows to pass by and thus to avoid encounters more easily than the traditional manure scraper that runs across the entire width of the walkway. Cows have, therefore, the chance to keep distance from the machine and need not step over it. It is further likely that the slow working speed of the robot (4.0 or 5.5 m/min) is advantageous for the cows’ ability to cope with the new situation and to avoid encounters with the machine.

Behavioral data support the assumption that habituation in cows has not yet been finalized since modifications in behavior continue until the end of the study. Based on these data, the evidence for a sensitization process is weak because the increasing time budgets related to lying and feeding, and the decreasing time budgets related to standing are neither significant nor consistent over the entire experimental period.

### Concentration of Cortisol Metabolites

We analyzed glucocorticoids in feces together with cardiac activity and animal behavior to investigate possible stress responses in dairy cows to robot scraper operation. The experimental period did not affect the cortisol metabolites concentration and therefore provided no evidence for a stress reaction in animals. In agreement with our investigation, a previous study analyzing potential stress responses caused by the milking robot did not identify any differences in the adrenocortical activity between cows milked in the milking robot and in the milking parlor (30). A more recent investigation detected effects of overstocking in the cortisol metabolites levels of primiparous cows but not in multiparous cows (31). Finally, a preceding study reported that under conditions of chronic stress, the hormonal status of animals mostly remains the same and recommended a more comprehensive consideration which integrates not only the concentration of cortisol metabolites but also changes in cardiac activity and behavioral and pathological data (45).

Previous studies showed that the cortisol metabolites concentration not only reflects the physiological and psychological states of the animal but also the diurnal and seasonal rhythms of glucocorticoid secretion, including ambient temperature and humidity. Variation in cortisol metabolites also occurs among individuals, species, and breeds. Moreover, glucocorticoids are important for modulating the function of the central nervous system and of energy metabolism (47–49). The randomized research design and the choice for analyzing the 11,17-DOA concentrations in the present study aimed to minimize these influencing factors. Fecal cortisol metabolites concentrations mirror the cortisol level secreted and eliminated over several hours; the influence of pulsatile secretion patterns and short-term fluctuation on the cortisol secretion is not as pronounced as in blood samples (33, 34). Since evaluation in cattle showed only little diurnal variation in the fecal metabolites concentration (50), diurnal rhythms play no role for the measurement of this parameter.

During the 4-week period under consideration, the stress hormone release in cows showed some indication for sensitization. Although insignificant, the rising cortisol metabolites concentrations imply that they will further increase and point to sensitization toward the repeated stimulus. Further research is needed to provide information about the cows’ response in the long term.

## Conclusion

The findings of our study demonstrated that in the first week after introducing the robot scraper, HRV was reduced in the cows, whereas lying and feeding were increased during robot scraper operation. We hypothesize that modification in animal behavior resulting in the reduced utilization of walkways subsequent to robot scraper introduction contributed to physiological stability and the attenuation of the impact of the stressor on cardiac function. Animals employed behavioral adjustments in order to overcome the stressful situation. The level of the cortisol metabolites remained constant during the entire experiment. Ongoing processes of habituation and sensitization cannot be ruled out due to the progression of behavioral parameters over the experimental periods. Thus, the outcome of this study suggests that the dairy cows after an initial phase of stress response were mainly capable of coping with the robot scraper and of adjusting to the new environment. It can be concluded that the cows’ stress response and impairment of animal welfare in the presence of the robot scraper are on a low level.

It is vital to understand the impact of autonomous mobile technology on dairy cows since exposure to stress impairs animal welfare, health, and performance. Additional studies can provide further insights into how the adaption process progresses. An open question is whether behavioral change will be reversed after a period of habituation and, if not, how to assess continuously altered behavioral patterns in terms of stress experience. The influence of robot scraper operation on the cortisol release beyond the period in question is also an issue, especially because cortisol was characterized as a long-term indicator of stress. It is further important to get a full picture of the mutual influence of both the different regulatory mechanisms – cardiac function, animal behavior, and adrenocortical system – and the long-term consequences of using an autonomous robotic cleaning system on dairy farms. Understanding the impact of the autonomous technology on animals is crucial in order to optimize robot scraper operation and to develop appropriate strategies that minimize possible adverse effects on cows and improve their welfare.

## Author Contributions

RD designed the experiment with the assistance of HB and EM. RD and HK carried out the data collection. RD, CL, and HK analyzed the data. EM and HK contributed reagents and analysis tools. RD and CL wrote the paper. EM, HK, and HB critically reviewed and commented on the manuscript.

## Conflict of Interest Statement

The authors declare that the research was conducted in the absence of any commercial or financial relationships that could be construed as a potential conflict of interest. The authors further declare that there are no patents that apply to the methods used.
